# Distribution of Arsenic and Risk Assessment of Activities on Soccer Pitches Irrigated with Arsenic-Contaminated Water

**DOI:** 10.3390/ijerph15061060

**Published:** 2018-05-24

**Authors:** Nadia Martínez-Villegas, Abraham Hernández, Diana Meza-Figueroa, Bhaskar Sen Gupta

**Affiliations:** 1 IPICyT, Instituto Potosino de Investigacion Cientifica y Tecnologica, Division de Geociencias Aplicadas, Camino a la Presa San Jose No. 2055, Col. Lomas 4a Sec., San Luis Potosi 78216, SLP, Mexico; abraham.hernandez@ipicyt.edu.mx; 2 Departamento de Geología, Universidad de Sonora, Rosales y Encinas s/n, Col. Centro, Hermosillo 83000, Sonora, Mexico; dmeza@ciencias.uson.mx; 3 School of Energy, Geoscience, Infrastructure & Society, Institute for Infrastructure and Environment, Water Academy, Heriot-Watt University, EGIS 2.02A William Arrol Building, Scotland EH14 4AS, UK; B.SenGupta@hw.ac.uk

**Keywords:** arsenic, water, soccer fields, soil, irrigation, risk characterization

## Abstract

The aim of this research was to estimate the risk of human exposure to arsenic due to sporting activities in a private soccer club in Mexico, where arsenic-contaminated water was regularly used for irrigation. For this purpose, the total concentration in the topsoil was considered for risk assessment. This was accomplished through three main objectives: (1) measuring arsenic concentrations in irrigation water and irrigated soils, (2) determining arsenic spatial distribution in shallow soils with Geographical Information Systems (GIS) using geostatistical analysis, and (3) collecting field and survey data to develop a risk assessment calculation for soccer activities in the soccer club. The results showed that the average arsenic concentrations in shallow soils (138.1 mg/kg) were 6.2 times higher than the Mexican threshold for domestic soils (22 mg/kg). Furthermore, dermal contact between exposed users and contaminated soils accounted for a maximum carcinogenic risk value of 1.8 × 10^−5^, which is one order of magnitude higher than the recommended risk value, while arsenic concentrations in the irrigation water were higher (6 mg/L) than the WHO’s permissible threshold in drinking water, explaining the contamination of soils after irrigation. To the best of our knowledge, this is the first risk study regarding dermal contact with arsenic following regular grass irrigation with contaminated water in soccer pitches.

## 1. Introduction

Arsenic (As) is a natural element present in the earth’s crust, which is widely distributed in the environment. It is present in the air, water, and land [1]. The major anthropogenic causes of the presence of As in the environment are mining and smelting activities [2]. Arsenic concentrations in uncontaminated natural soils are generally below 10 mg/kg [3], but As concentrations in natural topsoil irrigated with As-rich water show large variations depending on water and soil characteristics [4,5,6,7,8,9,10]. An As concentration of 22 mg/kg in soil used for residential and/or commercial purposes is considered safe, according to the Official Mexican Standard (NOM-147-SEMARNAT/SSA1-2004), which establishes criteria for the characterization and determination of soil remediation concentrations contaminated by As and other metals [11].

Arsenic is generally enriched in the fine fraction of soils [12]. In aerated soil, As is strongly retained by adsorption on iron (III) oxyhydroxides as well as other soil constituents, such as organic matter [13,14] and clay minerals with surface and edge charges [15]. In calcareous and gypsic soils, however, As may show high bioaccessibility [16], as it adsorbs and/or precipitates on either calcite or gypsum [17,18,19,20,21,22,23]. Pollution of soils due to the release of soluble As in water that is used for land irrigation is a major concern in areas located under arid or semi-arid conditions, such as the Comarca Lagunera [24], Zimapan [25], and Matehuala [16] in Mexico, Antofagasta in Chile, and the Chaco-Pampean area in Argentina, Pakistan, India, and China [26,27,28,29]. Environmental release of As from the irrigation water may result in transport to different media following the most common pathways such as: (i) transport to the subsurface and to groundwater by infiltration and (ii) retention in soils and further release in the atmosphere through suspension processes or to plants by bioaccumulation. A less studied exposure route is the physical contact of human skin with contaminated media.

For humans, the routes of exposure to As, a known carcinogen, are oral, inhalation, and dermal [30]. Common As exposure routes such as ingestion, physical contact, and inhalation represent a risk to health. This risk will be greater or lower depending on different parameters, such as the natural tolerance of the population, the specific characteristics of the element in question, the concentration in which it is found, and the duration of the exposure [31]. Arsenic, being a cumulative poison, may trigger adverse health effect with time in cases of chronic exposure [31]. However, the dermal route is not usually of concern [30]. Most published papers are related to the oral or inhalation exposure routes, and there is a paucity of research regarding dermal exposure routes [32,33,34]. Dermal exposure includes skin contact with water and soil. While the dermal absorption rate of soluble As in solution accounts for 4.8 ± 5.5% of total As, the absorption of As from soil and contaminated residues is negligible [35,36]. Yet, the skin is a critical organ for As toxicity because of local absorption and binding to sulfhydryl group-containing proteins after dermal exposure [37,38].

The contamination of soil with As via irrigation poses a carcinogenic risk of As exposure through dermal contact with the contaminated soil. The most popular sport in Mexico is soccer, and most public pitches are commonly occupied during weekends and weekday afternoons. Risk assessment is a complex process that requires the application of diverse tools, and one of the most important tasks is to observe the distribution of the contaminants under study [39]. Geographical Information Systems (GIS) provide tools that allow to observe, identify, and zonify the spatial distribution of contaminants that can create potential risk scenarios. GIS have been widely used to understand the spatial distribution of As in groundwater and soils [40,41,42]. In particular, inverse distance weighted (IDW) interpolation has been applied to create maps of As distribution in soils [43]. In this work, GIS was used to understand the spatial distribution of As in the topsoil from soccer pitches.

The aim of this research was to calculate the risk of human exposure to As due to sporting activities in a private soccer club in Mexico, where the soccer pitches are regularly irrigated with As-contaminated water. This was accomplished by: (1) determining As spatial distribution in shallow soils with GIS geostatistical analysis, (2) measuring As concentrations in irrigation water, and (3) developing a risk assessment calculation for soccer activities in the soccer club on the basis of field and questionnaire data. To the best of the authors’ knowledge, this is the first risk assessment study on dermal absorption of As from soccer pitches.

## 2. Materials and Methods

### 2.1. Study Site

A soccer club, known as Joya Verde sport club (2619149.76 m N, 337776.66 m E), is located in Cerrito Blanco, Matehuala, San Luis Potosi, Mexico. The club covers a total area of 47,000 m^2^ and is surrounded by semidesert vegetation and non-cultivated cropland. The club is approximately 14 years old and was built on communal land used for agricultural purposes between 1974 and 2003. Previous studies showed that water in the study area had extremely high concentrations of As (up to 19 mg/L) that derives from the dissolution of metallurgical wastes from an old abandoned smelter upstream, in the center of Matehuala [16,44,45]. Unfortunately, this contaminated water is the one and only concession of water granted to the Cerrito Blanco community for agriculture [16]. Besides As, no problems have been reported with other trace elements in surface and/or groundwater in the Cerrito Blanco area [44,46]. Because of the irrigation of soils with As-contaminated water, it is believed that this regular activity might have caused a gradual buildup of As in the soil.

The soccer club consists of irrigated areas (IA), comprising three different soccer pitches, half hectare each, and vegetated areas, referred to as non-irrigated areas (NIA) that surround the soccer pitches. Soccer pitch one was grass-seeded early in 2003, using two types of seeds, Bermuda or Grama (*Cynodon dactylon* (L.) Pers.) and Kikuyo or Coarse Grama (*Pennisetum clandestinum*). Pitch two was top-dressed with carpet grass, while pitch three was established in 2013 using patches of carpet grass that were removed from field number one to dress field number three. During the summer season, soccer pitches are mowed every eight days, while, during the winter, they are mowed every two months. Pesticides and chemical fertilizers are not used in these fields, however manure from subsistence agriculture is discretionarily applied in areas with poor grass cover. Each field is irrigated twice a week for 30 min at a rate of 7.63 L/s using surface water pumped from a channel known as Matehuala–Cerrito Blanco hydraulic complex.

**Figure 1:**
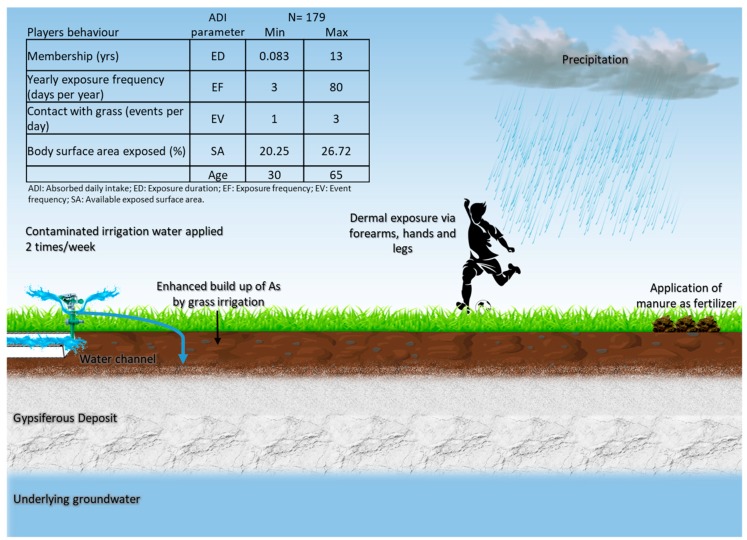
Refined conceptual model showing the pathway of concern with respect to As contamination: dermal absorption via contact with soil. Soccer statistics are based on 179 questionnaires acquired out of 201 from members of the soccer club under study.

On-site soccer activity is primarily dependent upon the local climate and time of the year. In this area, the temperature is 20.3 °C on average, January being the coldest month (14 °C) and June the hottest month (24 °C) [47]. Herein, precipitation accounts for 400 mm a year [47]. In the soccer club, eight to nine soccer games are practiced every Sunday. On average, 200 individuals visit the soccer club on a weekly basis. Most of the players are men above 45 years of age, who visit the place once a week. A conceptual model depicting the input of As into the fields, respective receptors, and the potential pathways of exposure is shown in Figure 1.

### 2.2. Soil and Surface Water Sampling and Analysis

As mentioned before, previous studies have shown that water in the study area had extremely high concentrations of As (up to 19 mg/L) over time [16,44]. In order to corroborate water As contamination, two surface water samples from the Matehuala–Cerrito Blanco channel and an irrigation faucet were collected in triplicate in acid-washed bottles. Irrigation water samples were filtered (<0.45 µm) and acidified to pH < 2 using HNO_3_. The samples were hermetically sealed and stored at 4 °C until analysis. Parameters such as temperature, pH, redox potential, electrical conductivity, total dissolved solids, oxidation–reduction potential, and dissolved oxygen were analyzed in situ in the irrigation water using a HI9829 multimeter from HANNA. Alkalinity was measured with a HACH alkalinity test kit model AL-DT.

**Figure 2:**
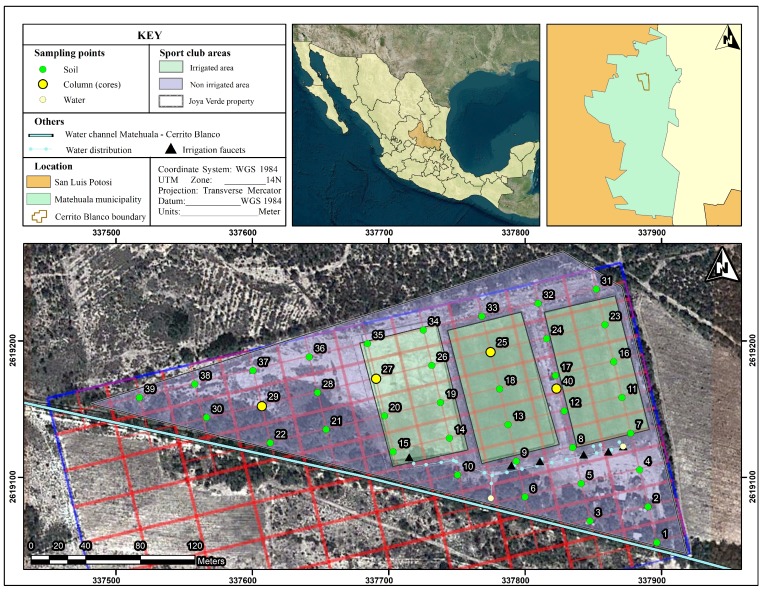
Site and sampling locations.

A total of 39 surface (0–5 cm) soil samples were collected with an auger from the IA (i.e., soccer fields and grass-off zones between fields) and the NIA (bare soil and scrub areas) using a systematic, stratified sampling technique based on a statistical method [48], as shown in Figure 2. Four soil cores (0–60 cm) were collected across both areas, IA and NIA, to determine As concentrations at various depths, according to the Mexican regulations and recommendations (NMX-132-SCFI-2001) [48]. Each soil core was divided into six segments of 0–10 cm, 10–20 cm, 20–30 cm, 30–40 cm, 40–50 cm, and 50–60 cm. 

All sampling locations were geo-referenced with a Garmin Etrex Personal navigator GPS unit. Duplicates were collected for every fifth sampling location for data quality purposes to make a total of 77 surface soil samples and 32 column samples. All soil samples were dried at room temperature and sieved to obtain the fraction less than 2 mm. Then, the soil samples were digested according to the slightly modified ISO 11466:1995 method. A sample of 1.0 g of soil was placed in a beaker, and then 10 mL of aqua regia (HNO_3_:HCl, 3:1) was added. This digestion procedure is adequate for analyzing total recoverable heavy metals in soils [49]. Residual elements, which are considered unimportant for estimating the mobility and behavior of elements of environmental interest, are not released by aqua regia digestion [50]. In this study, the As recovered by aqua regia digestion was called total As (tAs). The beakers were heated at near boiling temperature (≈85 °C) until the digests were evaporated to near dryness. The residues were redissolved in 10 mL of aqua regia, allowed to cool, transferred to volumetric flasks, brought to a final volume of 50 mL with 0.5 M nitric acid, filtered (through a Watman filter paper No. 40), and stored at 4 °C until analysis. Additionally, eight soil samples were randomly selected for texture determination by the Bouyoucos Hydrometer Method [51].

Total As determination was performed in both water and soil samples by inductively coupled plasma optical emission spectroscopy (ICP-EOS) [52]. Calibration with reference samples and blanks and replicate analysis for quality control were carried out to ensure the reliability of the analytical data. The calibration curve was in the range of 0.05 to 20 mg/L and showed good linearity (r = 0.999, *n* = 6). The detection limit was 0.10 mg/kg.

### 2.3. Spatial Analysis Using GIS

**Figure 3:**
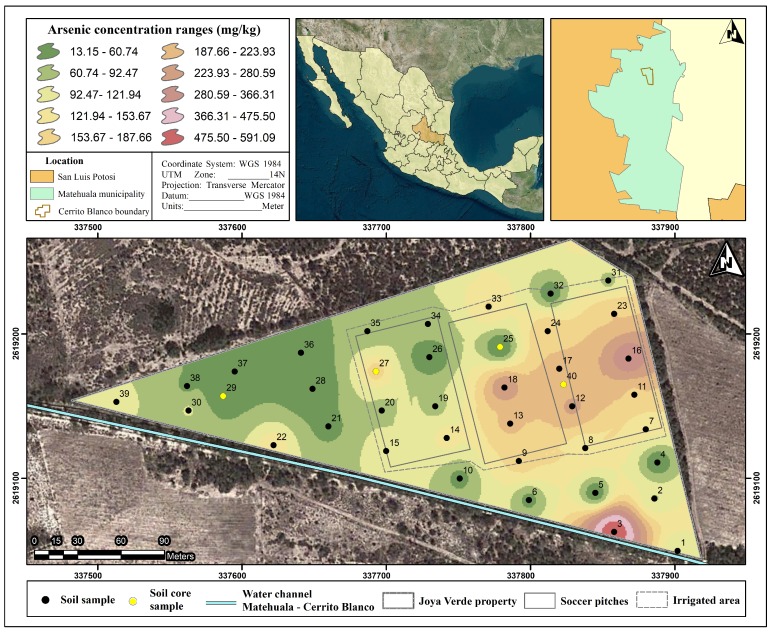
Geochemical map of tAs concentration in the surface soils by inverse distance weighting (IDW). Sampling locations and irrigated and non-irrigated areas are shown.

The sampling locations were plotted for the study site, and a geochemical map depicting As distribution in surface soils was generated using a GIS software (ArcMap 10.2.2, Environmental Systems Research Institute, Inc., Redlands, CA, USA). GIS has been widely used to understand the spatial distribution of As in groundwater and soils [39,40,41] and to represent, identify, and plot the spatial distribution of As in topsoil from soccer pitches. For interpolation, inverse distance weighting (IDW) was used to examine the distribution of As in the sports club surface soil, because (*i*) it is an appropriate method for interpolating regularly spaced data [53], (*ii*) it represents the real values at each specific sampling point of data, and (*iii*) it leads to smoother, gradual, and more precise representations of the data, taking into account the local variations [32,54,55,56,57]. The geochemical map obtained was overlain with a polygon depicting the outline of the soccer club (Figure 3).

### 2.4. Risk Characterization

Table 1 shows the equations and parameters used to characterize the potential on-site carcinogenic risk through the pathway of dermal absorption from As-contaminated soil according to the methodology for risk assessment proposed by the United States Environmental Protection Agency (USEPA) [58]. Field and laboratory analyses provided a set of values for the tAs concentration in sports club soil (C). Out of 201 questionnaires distributed to the players at the club, information regarding players’ habits on the soccer club was obtained from 179 responses. The respondents were requested to supply the following information: (1) “When you play, what do you usually wear (long-sleeve t-shirt, short-sleeve t-shirt, soccer pants, soccer shorts, long socks, short socks)”; (2) “How many games do you play per visit?”; (3) “How many times a year?”; (4) “How many years have you been visiting the club?”. The responses provided a range of values for the following input parameters: (1) exposed body parts (SA), (2) daily contact frequency with grass and soil (EV), (3) yearly exposure frequency (EF), and (4) lifetime exposure duration (ED). Monte Carlo simulation was used to account for the natural uncertainty and variability within a risk assessment [32]. The method derived a set of possible outputs for the site-specific exposure parameters described above, that were observed to have a range of values (Table 1). As the frequency plots of the parameters (C, EV, EF, and ED) were not normally distributed, the original data distributions were taken into account for the calculation of risk. By using the original distributions of the data, we expected a more realistic site-specific exposure time history of any player. Monte Carlo simulation was performed using the Oracle Crystal Ball complement for Microsoft Office Excel 2013. The software platform Oracle Crystal Ball is one of the most commonly used Monte Carlo modelling tools [59]. The number of iterations for the equation was set to 10,000. Input parameter values based on published data were used for dermal slope factor (SF), resident soil adherence factor (AF), absorption factor for As (ABS), conversion factor (CF), available exposed area (SA), body weight (BW), and averaging lifetime for carcinogens (AT) (Table 1).

(1) Risk = ADI × SF

(2) $$Where\;ADI\;=\frac{\;C\;\times \;AF\;\times \;ABS\;\times \;CF\;\times \;SA\;\times \;EV\;\times \;EF\;\times \;ED}{BW\;\times \;AT}$$

## 3. Results and Discussion

### 3.1. Arsenic Concentration in Irrigation Water

Table 2 shows the physicochemical characterization and concentrations of As in irrigation water. Arsenic concentrations were 6.7 ± 1.6 mg/L and 6.6 ± 0.2 mg/L in the samples collected from the channel and the faucet, respectively. These values exceeded the WHO’s permissible drinking water standard and were 15 times higher than the Mexican threshold for As in national waters (0.4 mg/L) [60]. These concentrations of As agree with previous studies [44,45,46] and are the result of metallurgical activities practiced in the area during the first half of the 20th century.

### 3.2. Arsenic Concentration in Soils

The tAs concentration of the surface soils in the study area ranged from 13.1 mg/kg to 591.3 mg/kg, with a mean concentration of 119.4 ± 109.5 mg/kg (*n* = 39) (Table 3). This mean value is higher than the background level reported for the area (4 to 35 mg/kg) [61], suggesting that irrigation with As-contaminated water might have increased the concentration of As in the soils. High background As levels have been attributed to a geochemical anomaly in the As content in the area [61]. Nevertheless, as mentioned above, As-contaminated water is applied to the soccer fields twice a week, and it is therefore estimated that, in the worst-case scenario, when no As leaches from the soil, 9.5 kilograms of As are added to the soccer fields per year, which accounts for nearly 150 kg of As added to these soils during the last 13 years, assuming an average concentration of 6 mg/L in the irrigation water.

Generally, the IDW output supported these observations (Figure 3), showing that the area that received the highest amount of irrigation had the highest concentration of As. Soil As concentrations were higher than the 22 mg/kg threshold for As in Mexico for domestic soils [11], suggesting a potential risk for humans. In the IA, tAs concentrations ranged from 13.1 mg/kg to 353.3 mg/kg, with a mean concentration of 138.1 ± 82.9 mg/kg, while in the NIA, tAs concentrations ranged from 13.5 mg/kg to 591.3 mg/kg, with a mean of 92.5 ± 137.9 mg/kg. In the NIA, however, an observation different from other observations was made. This observation corresponded to sampling point 3 with 591 mg/kg of As. As sampling point 3 scored beyond the upper outer fence (Q_3_ + 3IQR) of a box plot, where Q_3_ and IQR are the upper quartile and the interquartile range, respectively, it was assessed as an extreme outlier [62], with Q_3_ and IQR values of 169.61 and 120.28 mg/kg, respectively. Furthermore, as the observation was the result of an extraordinary event that does not represent any sampling area (IA or NIA) and belongs to a different setup (small subsistence farm) within the sport club, sampling point 3 was omitted from the NIA dataset. Outliers coming from different populations can reasonably be omitted from the data [62]. Furthermore, as sampling point 3 is within a fenced area restricted to authorized personnel only at the boundary of the NIA, it should not impact soccer players and/or their risk. Therefore, in the NIA, the mean As concentration decreased to 59.5 ± 37.7 mg/kg when removing the outlier. By taking the sample location into account, while removing the outlier, a significant difference (*p* = 4 × 10^−6^) between mean As concentrations in IA (*n* = 23) and NIA (*n* = 15) was observed on-site, with 138.1 and 59.5 mg/kg, respectively (Table 3). A close inspection of sample 3 showed, in addition to the high content of organic matter, the presence of copious white precipitates within the soil pores. Similar precipitates were observed in most of the IA samples as well as in the samples near to the channel. High organic matter and high As have also been observed in surface water in the area [44].

**Figure 4:**
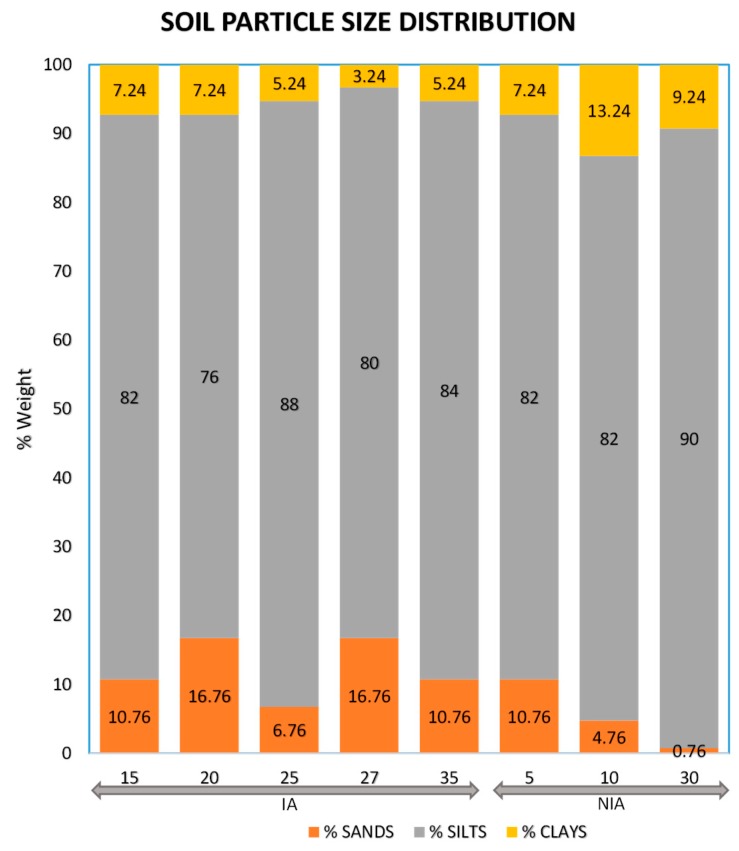
Soil particle size distribution (%) in eight samples collected from irrigated (IA) and non-irrigated areas (NIA) at the study site.

Soil fractionation showed that the bulk of both the IA soils and the NIA soils mostly comprised silt particles between 2 and 60 µm (Figure 4), indicating that soil texture was quite homogenous in the sports club. Particles below 200 µm represent a capture of 95% of the mass adhering to human skin [12]. Matehuala geology is represented by sedimentary marine rocks, which is part of the eastern physiographic province of the Sierra Madre Oriental [63]. Therefore, soils at the study area are largely dominated by either calcite or gypsum [16,44,64]. The stratigraphic sequence of Matehuala consists mainly of PreOxfordian, Upper Jurassic, and Cretaceous carbonated series, a little portion of Tertiary volcanic rocks that cover the city, and Quaternary alluvial material deposited on the Matehuala valley [63].

**Figure 5:**
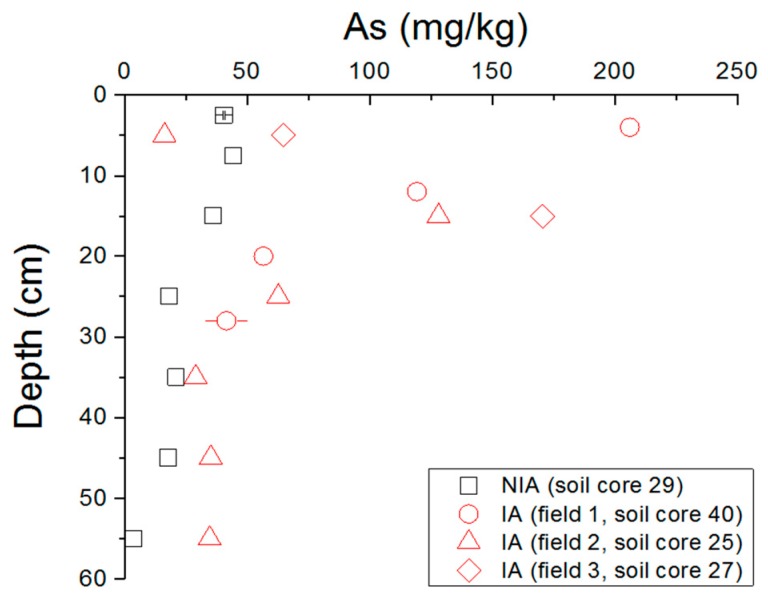
tAs in soil core samples from IA (samples 25, 27 and 40) and from NIA (sample 29).

In carbonate-rich soils, in which Fe and Al oxyhydroxide fractions are limited, As shows high transport [65], implying that, at the study site, As might migrate laterally and/or vertically. In order to examine the vertical transport of As on-site, four cores extending to the subsoil layer were collected along the IA and NIA. Because of the nature of soil and local geology, deeper cores could not be sampled. The results showed decreasing As concentrations with depth (Figure 5). The difference between the surface samples (0–10 cm) and the deeper samples (<10 cm) was greatest when the core was collected from IA, with differences ranging between 206.0 and 41.5 mg/kg for soil core 40 in IA and 44.37 and 3.61 mg/kg in NIA. The concentration of As in the surface soil sample of soil core 40 was 5 times higher than that of the subsurface soil sample (Figure 5), supporting the anthropogenic surficial source of As and exhibiting As leaching along the soil profile.

Questions remain on the processes that take place after addition of As to the soil and those that control the percolation of As downwards at the study site. While As is known to incorporate in calcite and gypsum by surface adsorption and/or (co)precipitation [17,18,19,20,21], high transport of As has been otherwise reported in carbonate-rich soils, in which Fe and Al oxyhydroxide fractions are limited [65]. Cycles of dissolution and precipitation of secondary arsenate minerals formed in soil after irrigation may also account for the accumulation of As in the topsoil and the vertical migration of As. Highly soluble arsenate minerals, such as haidingerite (CaHAsO_4_·H_2_O; *pk*_sp_ = 10^−4.79^) and pharmacolite (CaHAsO_4_·2H_2_O; *pk_sp_* = 10^−4.68^), in addition to guerinite (Ca_5_H_2_(AsO_4_)_4_·9H_2_O; *pk_sp_* = 10^−30.69^) have been found in the old abandoned smelter in Matehuala, as a result of the precipitation of As in a calcium-rich environment [64,66,67]. Previous studies have shown that As is highly soluble in water, and the more gypsum the soil contains, the lower the solubility of As, while higher calcite results in higher dissolution [16]. Although leaching of As to groundwater is a problem in contaminated sites [32,68], in this case, the silt soil texture and the presence of a hard subsurface gypsic horizon, found at 60 cm of depth, may reduce the soil’s ability to transmit water and help prevent the contamination of groundwater with As. Nonetheless, efforts should be made to contain the contamination in the upper 20 cm of the soil to protect a low-As shallow aquifer (<21 μgAs/L) [45] that runs NW to SE between 15 and 50 m [61,62,63].

### 3.3. Risk Assessment on Soccer Pitches

Soil As concentrations were higher than the 22 mg/kg threshold for As in Mexico for domestic soils [11], and a previous analysis has shown that there is a positive relationship between As content and fine-size particles [12], which represent the fractions that readily adhere to the skin [69] and thus can cross the dermis absorption barrier. Within a soccer club context, players can come into dermal contact with the soil and turf during soccer activities, through two primary routes; (i) leg–grass/soil contact when the player is practicing sliding tackles on turf soccer fields whilst wearing shorts and (ii) frequently, hand, forearm, and leg contact with turf and soccer ball on IA and NIA. The latter would be more common to all players during a normal game day. Although the authors acknowledge soil ingestion and the inhalation of fine dust particles as the main pathway for As exposure, the lesser studied potential risk via dermal absorption of As in a soccer field was examined in this study, where contaminated water is used for irrigation on a regular basis. A similar method was used previously on a golf course where seaweed fertilizer was applied to the turf [32]. The frequency and duration of contact and the degree of exposure are, in fact, greater for soccer players than golfers.

The risk of As exposure through the pathway of dermal absorption with contaminated soil is based on the assumption that As is in a form that may cause cancer (i.e., in an inorganic form) [56,70]. Such an assumption is fully reasonable for As in the irrigation water, in which As has been presumed to exist as arsenate species [44]. It may also be reasonable to propose As as an inorganic arsenate species incorporated in calcite and gypsum by surface adsorption and/or (co)precipitation, or precipitated in arsenate minerals in surface soils on-site [17,18,19,20,21]. A probabilistic risk method was used to calculate the best and worst-case carcinogenic risk values for soccer activities, based on the range of risk input parameter values (Table 1). These values were then compared to a published reference value for As to determine whether the risk is acceptable or not [58]. Yet, there is some discrepancy regarding the acceptable risk value for As exposure. The USEPA suggests that any risk value greater than 10^−6^ is unacceptable [58]. Such a value indicates that it is acceptable that one out of every 1,000,000 people die from cancer induced from exposure to As. However, other studies use an acceptable risk range of 10^−6^ to 10^−4^ for similar environmental health hazards [56,71,72,73,74], which indicates that within a population of one million, it is acceptable that one out of every 10,000 people die from cancer induced by exposure to As. The lack of an evidence-based acceptable risk threshold across a wide range of environmental contexts makes it difficult to ascertain whether a particular exposure scenario is hazardous or not. With respect to the irrigation of soccer pitches with As-contaminated water in this area, an uncertain scenario has occurred because of the transformation that As may undergo in the soil and turf to less toxic (in)organic forms and/or after dissolution on the skin (in sweat) of the layering of soil to more soluble/available forms.

In this study, the calculated risk of As exposure via dermal absorption was in the range of 1.0 × 10^−10^ to 1.8 × 10^−5^ (from Equations (1) and (2) and Table 1). With respect to the USEPA threshold, the maximum risk value observed at this site exceeded the threshold by one order of magnitude, indicating that the risk of As exposure to players at this study site is unacceptable. The results of the risk characterization indicated that the maximum soil As concentrations and the exposure scenarios did generate a carcinogenic lifetime risk that is higher than the acceptable risk level. Therefore, future increases in As soil content and in event frequency may result in higher exposure values. At the soccer club, soil particles have medium texture, consisting mostly of silts (Figure 4). The As applied to the soil via irrigation will accumulate in silt particles and in the topsoil (Figure 3 and Figure 5) [75]. This could, therefore, create a scenario where the risk of dermal absorption of As is greater than that observed. Furthermore, within the scenarios, exposure time, frequency of contact with soil, and fraction of body parts exposed may be greater than in our calculation. For example, during the windy season, which may last 60 days, usually starting during February, the exposed body parts and the frequency of contact between the soil and the skin would be greater, as the air contains dust during windy days, which may also favor increased soil ingestion as well as inhalation of soil particulates followed by ingestion after mucociliary clearance.

## 4. Conclusions

The findings of this study relate to the growing concern of As exposure risk to human health and contribute to the understanding of As enrichment and risk arising from domestic soil used for sports. The results showed that the mean As concentrations in soils in the soccer field examined (106.9 mg/kg) exceeded the Mexican threshold for As in domestic soils (22 mg/kg), with a significant difference (*p* = 7 × 10^−8^; *n* = 38). Higher concentrations of As were found in the irrigated soils (138.1 mg/kg; *n* = 23) than in the non-irrigated soils (59.5 mg/kg; *n* = 15), with a significant difference (*p* = 4 × 10^−6^). Arsenic concentrations in irrigation water were, likewise, higher than the WHO’s permissible threshold for drinking water, explaining, therefore, the contamination of soils after irrigation. Furthermore, risk calculations showed that the USEPA’s acceptable risk level for dermal contact with As through the soil was exceeded in this particular case. It is important to acknowledge the dermal risk of site users which, in turn, may be greater in scenarios of longer contact time. Other potential pathways of As exposure, such as fine dust inhalation and soil ingestion, may also increase the risk of As for soccer players’ health in the area and should be considered in further studies. Although the geological setting may suggest a low risk for As leaching to groundwater, As is likely to migrate deeply in the soil. Further investigation is required to explain the high As concentration found in soils in the small farm within the soccer club.

Recommendations derived from this study include: (1) for the players, the use of long sleeves and pants to reduced exposed body surface areas and showering immediately after exercising; (2) for the owner, the replacement of natural grass with artificial turf to avoid irrigation with contaminated water or irrigation with As-free water; (3) for the authorities, the provision of an As-free water source and/or the installation of an As treatment system, after which watering bans could be put into effect to restrict the use of As-contaminated water.

## Figures and Tables

**Table 1:** Risk characterization methodology according to USEPA (2004).

Exposure Parameter	Description	Value	Source
Risk	The probability of an individual developing cancer over a lifetime	-	Site specific
ADI (mg kg^−1^-day)	Absorbed daily intake	5 × 10^−6^	Site specific
SF (mg kg^−1^-day)^−1^	Dermal Slope Factor (based on a gastrointestinal absorption factor of 0.41)	3.66	RAIS (1998)
C (mg kg^−1^)	Concentration of As in soil	13.14–591.31	Site specific
AF (mg cm^−2^)	Resident soil adherence factor	0.01–0.08	USEPA (2004)
ABS	Absorption factor for As	0.03	USEPA (2004)
CF (kg mg^−1^)	Conversion factor	10^−6^	USEPA (2004)
SA (cm^2^)	Available exposed surface area	3300–5700	Site specific USEPA (2004)
EV (events day^−1^)	Event frequency	1–3	Site specific
EF (days yr^−1^)	Exposure frequency	3–80	Site specific
ED (yrs)	Exposure duration	0.083–13	Site specific
BW (kg)	Body weight	74.8	INEGI (2015)
AT (days yr^−1^)	Averaging lifetime for carcinogens	25,550	Agency for Toxic Substances and Disease Registry (2011)

**Table 2:** Physicochemical characterization of the irrigation water.

Sample	T (°C)	pH	EC (µS/cm)	TDS (mg/L)	ORP (mV)	DO (mg/L)	Alkalinity (mgCaCO_3_/L)	As (mg/L)
Channel	29.5	8.33	2567	1283	434.3	2.11	124	6.7 ± 1.6
Irrigation faucet	31.2	8.34	2601	1300	433.6	1.05	103	6.6 ± 0.2

T: Temperature; EC: Electrical conductivity; TDS: Total dissolved solids; ORP: Oxidation–reduction potential; DO: Dissolved oxygen.

**Table 3:** Total As concentrations (tAs) in surface soils in irrigated (IA) and non-irrigated areas (NIA).

Sampling Areas	*n* (Number of Samples)	Mean (mg/kg)	Std Error (mg/kg)	Min (mg/kg)	Max (mg/kg)
Whole sport club	39	119.4	109.5	13.1	591.3
Irrigated Area	23	138.1	82.9	13.5	353.3
Non-Irrigated area	16	92.5	137.9	13.6	591.3
